# Model organism futures in precision toxicology: tracking the emergence of a research repertoire

**DOI:** 10.1007/s10539-026-10009-9

**Published:** 2026-02-17

**Authors:** Rachel A. Ankeny, Sabina Leonelli

**Affiliations:** 1 Wageningen University, P.O. Box 8130, Wageningen, 6700 EW The Netherlands; 2 Philosophy and History of Science and Technology, Department of Science, Technology and Society (STS), Technical University of Munich, Augustenstrasse 40, 80333 Munich, Germany

**Keywords:** Toxicology, Precision toxicology, Model organisms, Bioremediation, Experimental organisms, Modelling, Scientific repertoires

## Abstract

This paper considers the continued use of simpler model organisms in newer forms of toxicological research in order to inform philosophical understandings of the epistemic roles played by such organisms in the contemporary life sciences. We focus on the emerging domain of ‘precision toxicology’ and consider three uses of model organisms within it, namely as (1) models of toxic effects and other forms of environmental exposures; (2) indicator species; and (3) bioremediators. We analyze the epistemic implications of these uses, arguing that they represent hybrid forms of modelling in comparison to traditional uses of model organisms, and identify similarities and differences between these emerging research practices and the model organism repertoire being adapted for use in this domain. Model organisms are simultaneously viewed as tools for intervention and representation within precision toxicology, in ways that differ from the model organism repertoire both in terms of the extent to which the models fit applied research goals and how they foster evolutionary and developmental understanding. Hence we argue that model organisms remain highly influential models in the life sciences but are being used in research more closely associated with the concept of ‘precision,’ and characterized by an ethos of intervention particularly in response to the environmental challenges associated to climate change and attention to the evolutionary and developmental grounding of health and disease. In closing, we reflect on the ways in which using the analytic framing associated with the repertoires approach facilitates the tracking of these developments in the contemporary life sciences. We also assess how they may affect the construction and significance of model systems over coming decades particularly in relation to precision-related research.

## Introduction

This paper considers the continued use of simpler model organisms (such as water fleas, fruit flies, and nematodes) in toxicological research and newer aspects of such uses, in order to inform philosophical understanding of the epistemic roles played by such organisms in the contemporary life sciences. We focus on the emerging domain of precision toxicology and consider three uses of model organisms within it, namely (1) as models of toxic effects and other forms of environmental exposures; (2) as indicator species; and (3) as bioremediators. We analyze the epistemic implications of these uses, arguing that they represent hybrid forms of modelling in comparison to traditional uses of model organisms, and identify similarities and differences between these emerging research practices and the existing model organism repertoire which is being redeployed in this domain. We reflect on the ways in which the analytic framework provided by the repertoires approach facilitates the tracking of these developments and provide an assessment of how they may affect the choice and significance of model systems over the coming decades, particularly in relation to precision-related research.

This paper has a second and closely intertwined goal, namely to provide an initial exploration of key concepts that warrant attention and additional analysis within the range of fields in which precision-focused approaches are increasingly common. Despite the rising prominence of precision science approaches, there has not been extensive discussion in the philosophy of science literature of these approaches beyond attention to precision medicine and health, nor has there been comparative analysis of fields that are rapidly developing under the banner of ‘precision.’ We argue that the repertoires framework previously proposed by us (Ankeny and Leonelli 2016) is uniquely suited to tracking the development of a field as a potentially distinct epistemic space (Müller-Wille and Rheinberger 2012) and community (Knorr Cetina 1999), and that it enables us to identify similarities (and differences) between fields focused on similar central concepts, in this case model organisms and also more broadly the concept of ‘precision.’

The notion of a repertoire is a heuristic tool intended to capture the conditions under which particular ways of doing research persist and expand beyond a specific project, resulting in a blueprint for scientific reasoning and practice in relation to specific goals, methods and (often interdisciplinary) forms of expertise (Ankeny and Leonelli 2016). In this paper, we focus on the evolution of the model organism repertoire in precision toxicology as characterized by the following features: a commitment to the multi-perspectival study of model organisms as referents for other species; substantive efforts to develop relevant know-how, expertise, protocols, instrumentation, and large-scale data collections; the production, use, and dissemination of standardized specimens and related infrastructures; an ethos of sharing data and techniques prior to publication; and reliance on long-term, blue-skies funding geared especially toward molecular approaches, similar to the model organism-associated sequencing projects (Ankeny and Leonelli 2020).

We discuss the ways in which this repertoire has been explicitly adopted by precision toxicology practitioners in response to recognized limitations of previous practices in toxicology, including its focus on rodents and on measuring toxicity levels with limited evolutionary understanding and ecological contextualization, and explore how the repertoire has been extensively modified to suit requirements and goals that are associated with precision-type approaches. As we demonstrate, precision toxicology has capitalized on many features of the model organism repertoire, while simultaneously changing the ways in which organisms are studied and used for environmental interventions, thus aligning its approaches with clear social and environmental needs while also expanding and deepening toxicology’s commitment to investigating fundamental biological questions. Precision toxicology also has attributed particular significance to model organisms as tools to conceptualize evolution, development, and ecosystems, and concretely affect them. In this domain, model organisms are viewed as hybrid tools for both intervention and representation, in ways that differ from the model organism repertoire (and previous practices in toxicology) in terms of the extent to which the models fit applied research goals and how the researchers intend to foster and expand evolutionary and developmental understanding. In the specific case of precision toxicology, we show that the model organism repertoire is evolving and being adapted for use in this domain, while also constituting a source of novelty and a radical departure from traditional ways of conducting toxicological research. We contend that this case demonstrates how model organisms remain highly influential in the life sciences particularly because of their association with the concept of ‘precision,’ and the grounding of the research in an ethos of intervention and attention to the evolutionary and developmental basis of health.

Note that in this paper we use the repertoires framework as a tool to track research developments ‘in flux’ without weighing in on whether precision toxicology as an emerging domain will develop into a fully-fledged and distinct scientific repertoire as it emerges and evolves. Indeed, our repertoires framework is qualitative and does not provide quantitative metrics for maturity, amount of distinctiveness or novelty compared to predecessor repertoires, or similar. But by studying shifts in the conceptualization and use of model organisms in toxicology due to the emergence of precision toxicology approaches, we demonstrate how the study of model species can go beyond its original uses and generate novel opportunities, in this case associated with precision-focused research and associated research repertoires.

Our account intertwines descriptive and normative analysis of scientific practices, grounded both on a close reading of published biological literature and collaborations with contemporary scholars in precision toxicology (e.g., thru discussions about their field and its future in the context of keynotes delivered by the authors at their meetings and those related to this topic, and work toward joint funding applications and publications). Central to our arguments is the idea that understanding how scientific models and concepts inform scientific understandings involves exploring how scientists use these models in practice, how those uses and associated arguments have changed over time, and what implications and significance these models have in relation to how we conceptualize life and thus how it is best studied. In this case as we show, the prioritization of precision approaches is associated with very particular understandings of how life is best studied.

## Organisms as models

Before delving into an analysis of the emerging field of precision toxicology, it is important to clarify the senses in which organisms can be viewed as ‘models’ according to the existing philosophical literature. Wimsatt (2000), Green (2013), and other philosophers have reflected on the multiplicity and complementarity of models in biological practices, features which become even more evident in applied fields such as precision toxicology as we will illustrate in our analysis. In any biological investigation, multiple models are mobilized simultaneously and jointly, raising issues about how best to coordinate and (where needed) integrate such models toward a common goal. Moreover, and most importantly for our purposes here, any one model can play multiple epistemic roles in ways that support the overarching aims of the research at hand and the concrete settings under which it takes place, as we will demonstrate using the case of model organisms in precision toxicology.

One key strand of literature has argued that models, and particularly model organisms, always perform a representational function by standing in for specific, typically wider groups of organisms and/or phenomena being explored (Ankeny and Leonelli 2020; see also Sartori 2023 for a formalization of this philosophical account with particular reference to justified inference). This approach is aligned with Roman Frigg and James Nguyen’s (2020) take on scientific models as quintessentially representational, insofar as they are defined by their capacity (in the eyes of researchers who use them) to plausibly and reliably have broad representational scope (in other words, the results of research with the organism can be projected onto a wider group of organisms: see Ankeny and Leonelli 2020), thereby making it possible to investigate and create knowledge about domains beyond the original source. This type of account also can be viewed as compatible with Michael Weisberg’s argument (2007) about the various relationships that models can have with phenomena in the world, recognizing that representational value does not imply a predefined, one-to-one diachronic relation between a model and the world, but rather that what a model represents can change depending on who is using it, in which setting, for which purposes, and via which scientific practices.

On the other hand, other philosophers have emphasized that models can work as tools for inquiry and intervention, in ways well characterized by the ‘models as mediators’ approach espoused by Margaret Morrison and Mary Morgan (1999). For example, Tarja Knuttila (2011, 2021) has rightly drawn attention to the artefactual quality of many models and the significance of constructing and manipulating these objects as ways to develop knowledge, while rejecting their representational features. Specifically with regard to model organisms, Weber (2004, 2014) views representation as sometimes present, but as a non-primary feature of model organisms given their diverse uses as tools to develop new experimental techniques, and argues as a result that they should not be viewed as actual models. Pierre-Luc Germain (2014) explores instrumental uses of model organisms while leaving open the questions of whether they should still be viewed as cases of modelling, or in fact how they might relate back to the representational power that may be attributed to such organisms.

Finally, detailed explorations by philosophers and historians of biology alike about specific examples of model organisms (e.g., Kohler 1994; Rader 2004; Leonelli 2007; Weber 2007; Nelson 2018) stress that model organisms are highly concrete and material entities^1^ and their physical features–as understood and deployed by researchers within specific conditions and through use of purpose-made instruments and certain settings–are crucial to their uses within scientific practices including manipulation, experimentation, and interpretation.

Against the background of these debates, a critical implication of our analysis in this paper is that viewing model organisms as artefacts or tools for intervention is not mutually exclusive with having representational functions. For instance, just as urban planners can use a scale model of a city both to capture local traffic patterns and explore alternative configurations of traffic flow, thereby developing proposals for future modifications, so too do molecular biologists use RNA segments both as representations of specific cell types and as probes for transcriptional profiling, and in many cases at the same time. We must look at actual scientific practices in specific research domains to determine how organisms (or any other models) are being used.

What is clear from the previous scholarship on model organisms is that their epistemic functions and significance are typically tied to their material properties and to the ways in which researchers choose to exploit or ignore those properties. Building on our previous work (Ankeny and Leonelli 2011, 2020), we contend that the instrumental role sometimes played by model organisms typically has representation at its core, since the properties of these concrete models as material objects strongly affects what they can be taken to stand in for and how they perform this representational function. Greater focus on the relationship between concrete manipulation (and other instrumental uses of models) and the representative function of models will allow us to better understand their use as tools for intervention. Our exploration of precision toxicology is proffered to provide just such an example.

Precision toxicology presents an ideal space in which to investigate issues associated with representing and intervening, and their potential interrelations in modelling practices, because of the wide-ranging sites in which it is practiced, including laboratories and real-world environments, and the diverse ways in which model organisms are used within its practices. In the cases we discuss below, we argue that the representational and instrumental functions of model organisms end up being deeply intertwined and even interdependent.

## What is precision toxicology?

Precision toxicology arose in part out of long-standing traditions in the field of toxicology, which is the study of the potential harmful effects of various substances such as chemicals, pesticides, food additives, and pharmaceuticals on biological organisms and the environment via acute or chronic exposures. Most toxicological research relied on use of non-human animals to establish whether certain agents are harmful to humans and at what dosage or exposure, thereby maintaining a relatively narrow, applied focus and keeping a distance from broader biological research and questions concerning the evolution, developmental trajectories, and morphogenetic traits favoring certain types of organic reactions. The key impetus behind toxicological research throughout the second half of the 20th century has been the exploration of organismal reactions to chemicals across diverse conditions, the measurement of which levels of exposure are likely to have, in most cases, harmful physiological effects, and the forms of therapy and response most likely to support recovery and resilience in humans (Woolf 2021).^2^ Questions about the mechanisms generating such effects, their evolutionary and developmental causes, and the ways in which they could be offset of course remained relevant, but were not explored in detail within this domain. Hence this field operated largely separately from more fundamental biological debates in part due to the need to be responsive to immediate, localized concerns and provide actionable solutions to the serious effects of chemicals on human populations across the globe (Davis 2014).^3^

Given the conditions of toxicological research, testing with humans is unwieldy and potentially unethical especially at higher doses. Among the key animal models utilized as surrogates for humans were more traditional rodent model organisms such as rat and mouse, as well as others commonly used in biomedical experimentation including dog, ferret, rabbit, hamster, guinea pig, primates, and minipigs (Gad 2006). More basic research occurs wholly within labs, with rodents exposed to certain chemicals whose effects are measured (King-Herbert and Vasbinder 2020). Rodents also feature particularly prominently in research focused on measuring environmental pollution levels, acting as indicator species for contamination as well as the potential biological effects of certain chemicals. This type of field research consists of the dispersion of rodents in the environment of interest and their subsequent retrieval (sometimes once they are killed off by toxins) for biochemical screening and examination, a strand of research that is extremely time consuming, expensive, and labor-intensive.^4^

Increased attention to the 3Rs of animal experimentation (replacement, reduction, and refinement), together with steeply rising costs due to the large number of animals required, resulted in efforts from the early 2000s onward to pursue other alternatives to mammalian models (de Bruin et al. 2009). Some researchers began to develop in vivo experimental systems in what came to be called the new approach methods (NAMs) movement which began to gain momentum (Ankeny et al. 2024). Significant changes in regulation and policy resulted (see, e.g., NTP 2004), particularly in the European Union related to chemical testing to reduce or eliminate the use of experimental animals, along with requiring risk assessment for chemicals not only in terms of impacts on humans but also on wildlife and the environment more generally (see e.g. Dulio et al. 2018; Brooks et al. 2020; Naidu et al. 2021), and in more realistic and environmentally relevant concentrations than usually occurs in acute toxicity testing (Abdullahi et al. 2022a).

Thus some toxicological researchers began to explore expanded use of lower-level animal models such as invertebrates or lower vertebrates. These alternative animal models were viewed as potentially fruitful as they had many of the usual characteristics cited in discussions of organismal choice, such as being inexpensive, plentiful, and easy to manipulate in the lab, and having low maintenance costs, short life cycles, asexual reproduction, and high fecundity (Dietrich et al. 2020); however there also were concerns, notably about the likelihood of translatability of findings to higher animals and humans. These alternatives included some of the more standard model organisms such as zebrafish, nematodes, chick embryos, and *Daphnia*, but also brine shrimp *(Artemia)*, rotifers, mollusks, and copepods (planktonic crustaceans) (Khabib et al. 2022).

Precision toxicology as a recognized field can be dated to the late 2010s, and explicitly formed in response to these forces and due to recognition in various quarters of the need for reassessment of long-standing practices in toxicology. Critics described the ad hoc manner in which they had developed, resulting in a ‘cumbersome’ system (Krewski et al. 2010) plagued by substantially different requirements for chemical testing and generation of data that did not provide adequate answers regarding risks to human health. Many chemicals had never been tested despite potential human exposure to them: a recent analysis documented over 350,000 chemicals or mixtures registered for production and use around the world that have little to no toxicological data associated with them (Wang et al. 2020). In addition, pressures were mounting to develop effective interventions to counter toxic effects on humans and the environment, especially given the abundant opportunities and strong need for such technologies on our planet which is so massively overpopulated by chemicals created and dispersed by human activities (Landecker in press).

The terminology of ‘precision’ has diverse meanings in the various fields in which it is currently utilized, including medicine and health, agriculture, and even cosmology. Its use typically emphasizes more data-intensive and large-scale practices, enabled by technological progress in data collection and AI-led analysis and multi-scale integration, that permit closer examination of variability in the phenomenon of interest in order to foster targeted and impactful interventions (e.g., Polk et al. 2023). Thus these practices are in direct contrast to those that characterized the biosciences of the mid- and late 20th century, including early model organism research in molecular and developmental biology, which tended to focus on blue skies research to articulate biological fundamentals with limited applied goals in the short term and on generalizable commonalities without attention to context such as in the genomic sequencing projects of the 1990s which aimed to develop baseline data for key model organisms and for humans (Ankeny and Leonelli 2020; García-Sancho and Lowe 2023).

For example, precision medicine has been described as “an approach to disease treatment and prevention that seeks to maximize effectiveness by taking into account individual variability in genes, environment, and lifestyle” (NIH 2015). The notion of precision invoked here involves detailed measurements of contributors to disease including at the molecular and environmental levels, and more refined tools to collect and measure them, that in turn result in the development of interventions tailored to individual patients or finely grained sub-populations into which patients can be classified. Hence precision medicine is sometimes described as ‘personalized medicine,’ a concept and set of approaches which have been extensively explored and heavily critiqued in philosophy of science and beyond (e.g., Thompson 2013; Green et al. 2022; Prainsack 2017; Erikainen and Chan 2019; Green and Vogt 2022; Polk et al. 2023; Tabery 2023). There is an inherent tension or even paradox in precision medicine, namely that uncertainty is a key attribute of the practices associated with it (e.g., Hunter 2016; Kimmelman and Tannock 2018; Vogt 2022). Such uncertainty has been argued to be due not to the relative immaturity of the field or its associated technologies, but is a consequence of the underlying ontological, epistemological, and practical complexities associated with it (Lohse 2023).

Precision toxicology is a highly interdisciplinary and porous domain, with overlaps in its methods and key concepts with medicine, environmental science, marine biology, chemistry, and numerous other fields including all of the subfields of toxicology (including regulatory toxicology and other emerging subfields such as ecotoxicology: see Brooks et al. 2023; Henke et al. 2024), and its clear ambitions to bring together human and ecological toxicology to address concerns about environmental health (Colbourne et al. 2022). The earliest published approaches explicitly described as ‘precision toxicology’ emphasized high-throughput systems and had roots in high-throughput screening methods for drug discovery (Giacomotto and Segalat 2010) which used intact organisms to screen chemical libraries, with smaller and cheaper model organisms such as the nematode, the fruit fly, and zebrafish coming to be preferred to laboratory mice systems. This approach had roots in high-throughput screening methods for drug discovery (Giacomotto and Segalat 2010) which used intact organisms to screen chemical libraries, with smaller and cheaper model organisms such as the nematode, the fruit fly, and zebrafish coming to be preferred to laboratory mice. There also was use of single cell sequencing-based technologies to perform toxicological evaluations of drugs or chemicals on a small number or of or a single cell associated with a phenotype of interest (Zhang et al. 2017), building on ongoing growth of in vitro systems in toxicology more generally (Ghallab and Bolt 2014).

**Fig. 1:**
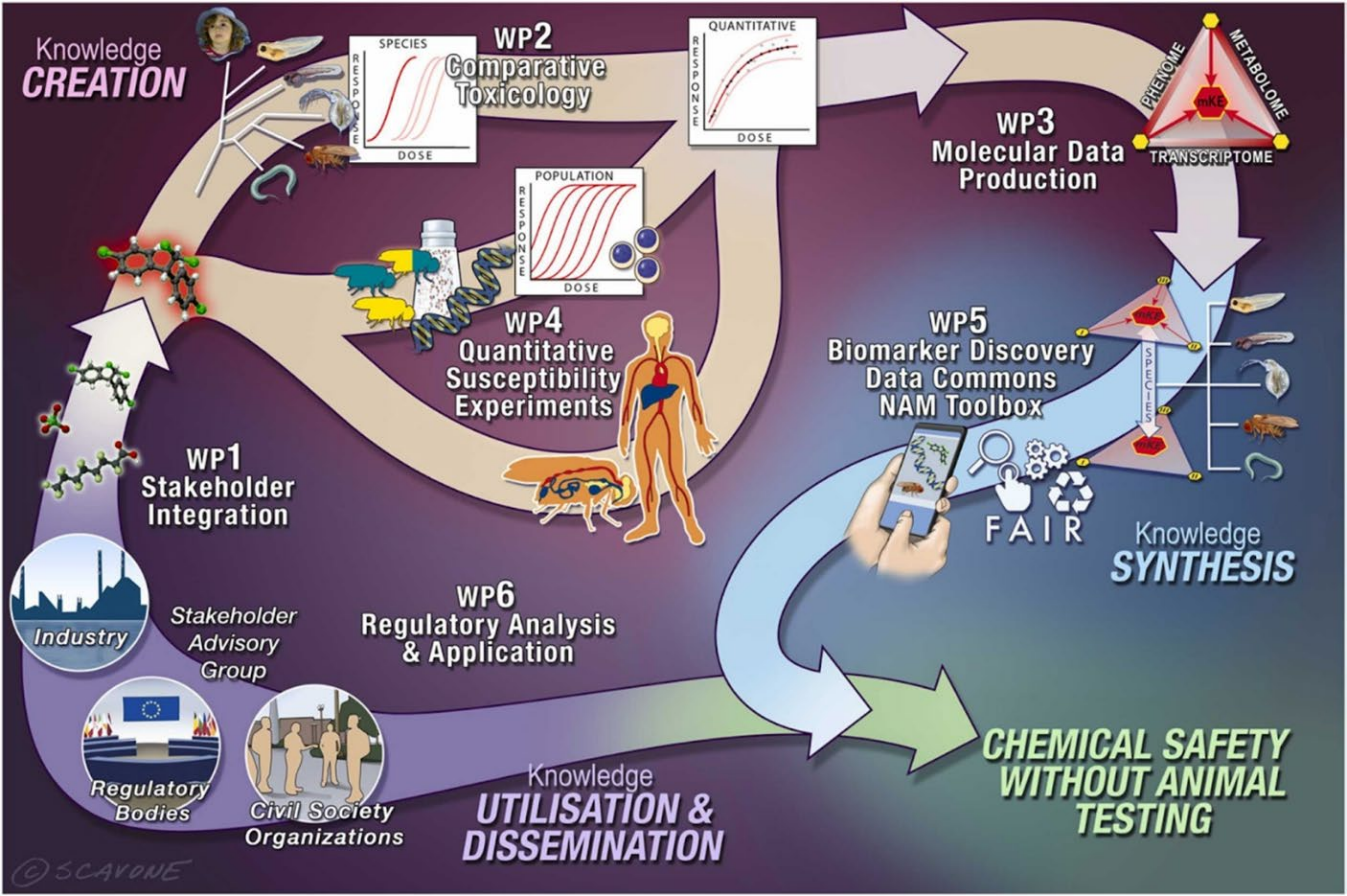
Reproduced from Colbourne and the PrecisionTox Consortium 2023 illustrating the components and proposed workflow of the PrecisionTox Initiative including six work packages (WP)

A major PrecisionTox consortium launched in 2021 focused on bringing together several fields, namely genetics, genomics, metabolomics, and evolutionary and developmental biological methods, to investigate the toxicity of chemicals and explore their actions with regard to the disruption of biological processes, particularly those tied to human health (Anonymous 2021). The goals of this initiative included those discussed above, namely developing alternatives to animal testing due to their slow pace, high cost, and associated ethical concerns, and producing clearer guidance for converting research findings into evidence-based interventions and policies to protect human health (Colbourne et al. 2023), given that standard laboratory animals such as rodents were recognized to be highly imperfect predictors of human toxicological response (Bailey et al. 2014) (Fig. 1).

But what distinguishes these precision toxicology efforts from other toxicological approaches before and parallel to them is the combination of several disciplinary approaches and its focus on what it terms ‘precision.’ In addition, its central goal is to identify conserved toxicity pathways and understand their significance to human health, including exploring variations in individual susceptibility that would permit more precision and accuracy in evaluating risk and setting exposure thresholds to protect even the most vulnerable or susceptible. Thus a suite of five model organisms (fruit flies, nematodes, and water fleas together with embryos of clawed frogs and of zebrafish) were selected to produce comparative data about the evolutionary origins of biomolecular interactions that are predictive of adverse health effects (Colbourne et al. 2023). Given the ever-increasing numbers of chemicals and growing demand for evidence-based regulation of hazardous substances, the importance of high-throughput approaches was clearly recognized, along with the potential of harnessing available genomic data and other resources associated with model organisms to measure not only risk thresholds but variations in susceptibility.

## Model organisms in precision toxicology

A key commonality found across so-called ‘precision’ sciences is the measurement of fine-grained variations and hence establish significant differences across the phenomenon of interest, which in turn permits interventions in targeted and site- or population-specific ways. Precision toxicology focuses on this strategy using model organisms such as fruit flies, nematodes, and water fleas, and also by capitalizing on what has previously been described by us as the ‘model organism’ repertoire (Ankeny and Leonelli 2016, 2020). Current proponents of precision toxicology note the need to study whole organisms in order to observe health-relevant changes involving interactions among different cell, tissue, and organ systems (Colbourne et al. 2023) rather than in vivo systems such as single cells. Hence model organisms are being utilized for high-throughput screening to reveal disease pathways and susceptibilities, and to assess the efficacy and toxicity of potential treatments.

In what follows, we discuss three typical and well-established uses of model organisms in toxicology and compare how these are approached and understood in contemporary precision toxicology: as indicator species, bioremediators, and models for humans. We show that in all uses, even the first two where the model organisms are being used as tools and interventions, substantive representational assumptions are required. Thus precision toxicology is harnessing the reasoning underlying traditional model organism research which relies heavily on model organisms’ representational functions in order to deepen biological understandings of the evolutionary pathways associated with toxicity. Hence the model organisms used have hybrid roles and are both instrumental and representational.

### Model organisms in precision toxicology: Models of toxic effects (1)

Model organisms have a long history of use as models for toxicity particularly effects on humans. Toxicity testing is expressly done with the expectation that information acquired in a particular model (often a rodent model organism) will apply to other biological systems, with each model having strengths and limitations depending on the questions under investigation (Hunt 2017). Mammalian laboratory animals have served as the ‘gold standard’ to date (on problematizations of this notion, see Ankeny et al. 2024), because they share similar developmental pathways and most organs with humans. However as noted above, only a small fraction of chemicals to which humans are exposed have been assessed. Further, rodent models have been found in metanalyses to predict specific toxic effects in humans only about 50% of the time (Hartung 2009; Knight et al. 2009). Hence researchers are developing alternative standardized testing approaches for a range of toxicological effects, and it is in this space that precision toxicology has gained particular traction.

For instance, the nematode *Caenorhabditis elegans* has been used as the basis for a relatively inexpensive and fast tracker for neurotoxicological impacts of chemicals on mammalian developmental processes (Hunt 2017). *C. elegans* has been argued to serve as a bridge between in vitro assays and mammalian toxicity testing, as many pathways of toxicity and modes of toxic action have been found to be conserved between worms and humans, based on our deep knowledge about their comparative genomic sequences and their functions associated with its long history as a model organism. More recently, *C. elegans* has been used to study one or a small panel of compounds, extracts, or nanomaterials; the effects of genetic background on potential therapeutic activity of compounds using *C. elegans* models of human disease; and the identification of pathways of toxic action for individual chemicals (Hunt et al. 2020). Hence in these types of uses, the worm is serving as a tool and hence has a strongly interventional role. However the basis of its validity to provide projectability to more generalized conclusions, and particularly those that apply to humans, is its representational role as a model.

An additional example can be found in Daphnia research. It had previously been thought that acquired tolerance to chemical stress (known as ‘experienced genotypes’) could be evolutionarily advantageous in cases of novel chemical stress as compared to ‘naive genotypes.’ However historical exposure to chemical stress has been found to cause reduced genome-wide diversity in Daphnia, leading to lower cross-generational tolerance to novel chemical stress underpinned by reduced diversity in detoxification, catabolism, and endocrine genes. At the same time, the experienced genotypes showed comparatively higher fitness when exposed to the same chemicals that had been recorded in the historical environment (Cuenca-Cambronero et al. 2018; Abdullahi et al. 2022a). Noting these tensions has prompted questions around the long-term effects of habituation to certain kinds of exposures for different organisms, which needs to be investigated further in light of these results. Precision toxicology approaches support such research due to their focus on uses of model organisms in high-throughput experimental set-ups, and the ability to analyze results in terms of norms and variations.

Hence precision toxicology approaches promote the idea that evolutionary history could be reframed in terms of susceptibility, for instance by tracking organisms’ changing capacities to react to chemical stressors and using those findings as a guiding thread for understanding and studying the history of life and of the interrelations among organisms, particularly in our modern chemically mediated world (Colbourne et al. 2022). Model organisms are thus used as models for the effects of toxicity levels (and other forms of environmental exposure) on more complex organisms as well as entire ecosystems due to their abilities to represent conserved genetic pathways. They are both synchronic models that serve as tools for phylogenetic mapping of organisms based on dissimilarities in how they respond to toxic substances, as well as diachronic models that serve representational roles which make it possible to analyze phylogenetic relations and conserved pathways over time through the lens of toxicity. 

### Model organisms in precision toxicology: indicator species (2)

A key use of experimental organisms within toxicology, with an illustrious and long history, is as indicator species or sentinels, such as canaries in coal mines or other organisms used to serve as early warning systems for toxic gases. A sentinel species is one that (1) is more sensitive than humans and most other animals to toxic exposures; (2) shares the same environment as humans; and (3) produces a readily detectable effect due to any toxic exposure. For instance a diverse range of fish that are non-model organisms (as they lack well-annotated genomic databases) are used as sentinel species for proteomic ecotoxicology studies (with nearly half of all studies using fish involving these species: see Henke et al. 2024) to detect contaminant stress under diverse environmental conditions including the freshwater fathead minnow *(Pimephales promelas)*, largemouth bass *(Micropterus salmoides)*, rainbow trout *(Oncorhynchus mykiss)*, and rare minnows *(Gobiocypris rarus)*, among many other species (Sanchez et al. 2011).

*Drosophila melanogaster* also has been utilized as a sentinel species. Proposals for this date back to the 1920s when fruit flies made their first appearance as a popular organism able to cross laboratory and field research (Kohler 1994) and were picked up again in the 1990s (e.g., Parsons 1991). Botanists interested in *Arabidopsis thaliana* (the thale cress that became the main model organism for plant science at the turn of the 1990s) have long regarded its ability to change leaf color to reflect the mineral composition of the soil as a significant and highly useful biological trait (Clemens 2001; Provart et al. 2016). This line of research has developed into using *Arabidopsis thaliana*, as well as related subspecies such as *Arabidopsis halleri*, as tools to identify pollutants (Cobbett and Meagher 2002; Corso et al. 2021) while also feeding much broader movements focused on the use of plants as environmental sentinels as exemplified by the International Plant Sentinel Network (Botanical Gardens Conservation International n.d.).

Use of a model organism as a sentinel species in precision toxicology also involves tracking specific reactions of the organism to various environmental conditions and particularly to substances present in the ecosystem, but aims to go one step further than traditional toxicology due to the preferred organisms which bring with them representational power particularly for projectability to humans. For instance over the last decade, many developments have occurred in *D. melanogaster* using molecular tools to understand and exploit epigenetic reactions to specific biochemical exposures and to develop standardized and reproducible protocols (Holsopple et al. 2023) including use of metabolomics to uncover adverse outcomes pathways using fruit flies (Rand et al. 2023). Using orthologs within conserved pathways can enable cross-species extrapolation and allows early diagnosis of the potential hazards of chemical pollution, even when chemicals are present at sublethal concentrations.

While the best-known model organisms have all been used as indicators for one type of pollutant or another in traditional toxicology, a species that deserves special attention in relation to precision toxicology is Daphnia. Daphnia have very short generation times and rapidly growing genomics resources (particularly *D. magna)* and have been found to share many ancestral gene families with humans, a feature that is critical for precision toxicology (Colbourne et al. 2011). *D. magna* is an important sentinel species because it also is a keystone species (essential to the function of its ecosystem) for freshwater ecology, which makes it a particularly powerful indicator (e.g., for water quality and freshwater food webs) (Abdullahi et al. 2022b). However it has significant representational features because of the ease of comparison and quantification of biomarkers especially at the molecular level due to the relative simplicity of creating clones. Having several individuals with the same genetic make-up provides a baseline against which variation can be more easily spotted, studied, interpreted, and projected back to humans.

### Model organisms in precision toxicology: leveraging bioremediation for new research directions (3)

Bioremediation involves processes that can affect the environment in ways that help to address the overabundance of specific substances, for instance by transforming or accumulating given chemicals or removing excessive nutrients. Arabidopsis has a long history of use in decontamination, capitalizing on the weed’s ability to metabolize metal ions in the environment, and thereby exploit the close connection between plants and the soil (Cobbett and Maegher 2002; Ojuederie and Babalola 2017). The plant’s capacity to multiply and spread quickly and effectively within a wide variety of sites, regardless of weather conditions, makes it attractive as both an indicator and a bioremediator. The characteristics of model organisms that have been traditionally favored because they enhance tractability in the lab, such as short life cycles, low cost, and ease of breeding and growth, are also privileged in this use in toxicology, but for different reasons, namely because of the opportunities that they offer for these organisms to infiltrate ecosystems with little effort and expense. These are, in essence, invasive species under most conditions, and this feature makes them well-equipped for use across different environments in a variety of locations (and thus across a spectrum of temperatures and humidity levels, among other factors).

Daphnia has been increasingly studied and used as a tool for bioremediation. For instance, Daphnia has been found to be an effective decontaminant for wastewater, even when compared to more well-recognized organisms widely used for that purpose such as algae and bacteria routinely deployed for waste management (e.g., Dell’Anno et al. 2021). Daphnia has been proven to reduce the presence and noxious effects on the environment of chemicals as diverse as pharmaceuticals such as codeine and naproxen, and industrial or agricultural by-products such as heavy metals (arsenic), biozide (atrazine), and industrial chemicals (including perfluorooctane sulfonate or PFOS).

**Fig. 2:**
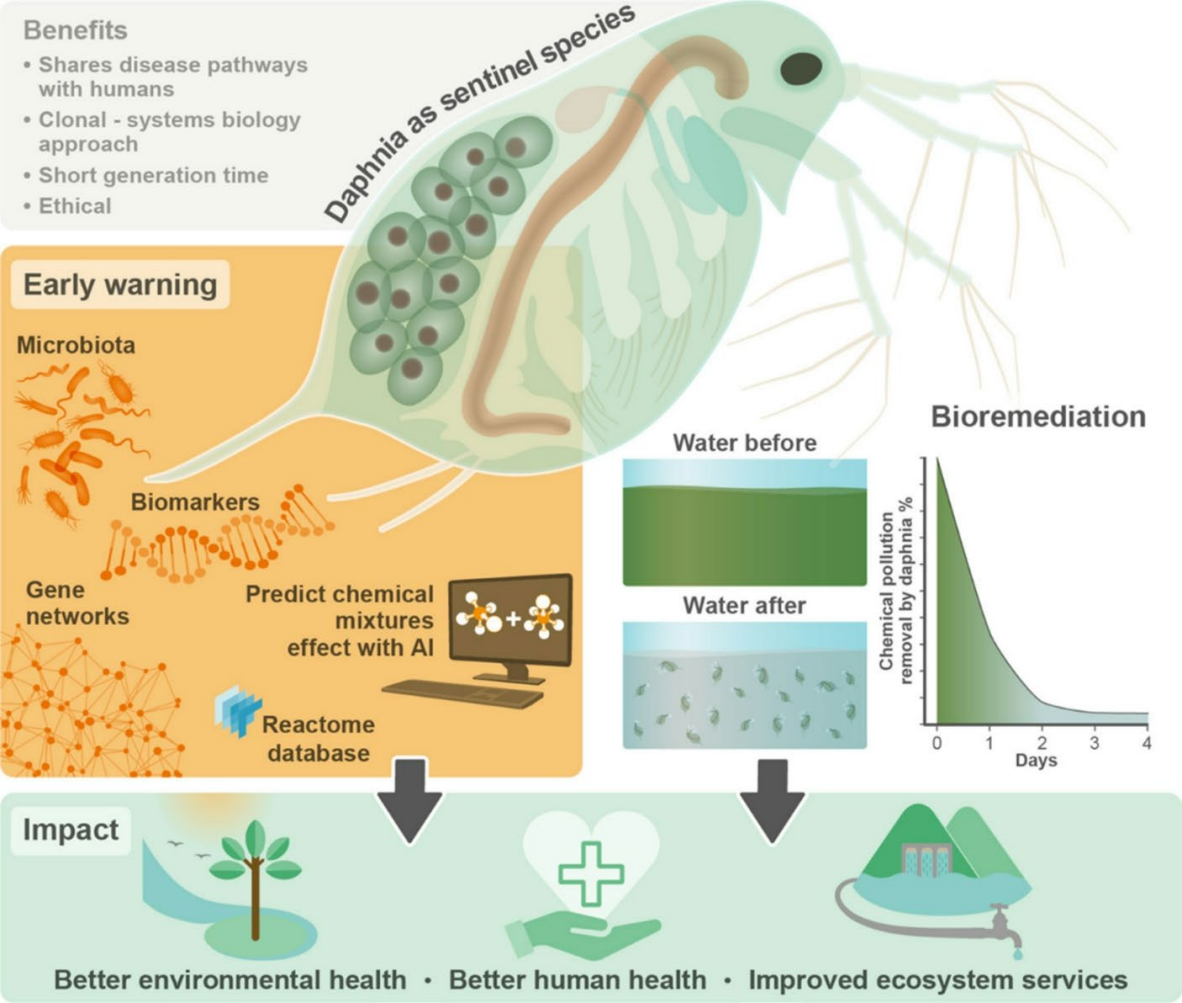
Reproduced from Abdullahi et al. (2022b) showing the use of Daphnia as both a sensor and a bioremediator

In precision toxicology, research using Daphnia as a bioremediator goes one step further (see Fig. 2): ‘omics’ technologies are applied to organisms being exposed to real-world chemical mixtures to measure exposure-induced biomolecular changes and link co-response networks of genes and metabolites to these chemical mixtures, including those where there are sublethal doses that would not be detectable using the usual toxicological approaches (Abdullhi et al. 2022b). Because of the use of high-throughput technologies, this approach is cost-effective way to generate recognizable signatures of chemical exposure that potentially reflect targets of toxicity. This strategy parallels those in precision medicine that seek to link molecular-level information to the health of a subject and hence represents a redeployment of precision technological approaches.

Advocates of precision toxicology approaches stress that these approaches differ significantly from traditional toxicology: *Daphnia* strains used as bioremediators serve as a natural experiment for studying real-life exposures to multiple chemicals, rather than relying on narrow, lab-based experimentation common in traditional toxicology which focuses on proving causal relationships between chemicals and certain (usually lethal) endpoints. In contrast, the precision toxicology approach uses advanced computational approaches such as machine learning together with the hypothesized evolutionary conservation of molecular pathways to identify correlations between chemicals within mixtures and putative molecular key events to in turn identify functional pathways even before significant harm occurs.

Perhaps most importantly, studies of the effects of toxic substances on humans and on the environment have typically been disconnected and compartmentalized because vertebrate models (such as rodents) are favored as surrogates for humans, whereas algal, invertebrate, and fish species including Daphnia that are ecologically relevant have been used primarily as sentinels (surrogates for the environment) and bioremediators. Thus cross-species extrapolation between human and environmental toxicity research has generally not occurred (LaLone et al. 2021). In contrast, precision toxicology stresses the potential for cross-species extrapolation due to shared evolutionary history (what they call “toxicity by descent”) including to humans. Thus it is argued that precision toxicology provides a more cohesive experimental design that permits quantitative identification of functionally conserved putative molecular key events among species and their links to chemical toxicity (Abdullhi et al. 2022b).

## Developing the precision toxicology repertoire

It should be apparent from our analysis of the three uses of model organisms in precision toxicology that this emerging field has built on approaches, findings, and technologies not only from toxicology, but also developed through early genomic and developmental biology research with model organisms. The representational target that characterized this form of modelling (of say humans) has been maintained and heavily leveraged. Does precision toxicology represent a significant change in how toxicological research is done, or simply an extension of what came before with a bit of new technologies thrown in? And how does the widespread emergence of model organisms as key models in this field relate to the practices and traditions underpinning model organism research? One way of reflecting on these questions is to consider what the broader sets of practices associated with this emerging field are, using what we previously have termed a ‘repertoire’ framework (Ankeny and Leonelli 2016). In this section, we discuss the features of model organism research (a quintessential repertoire in our view) that are being explicitly adopted by precision toxicology advocates and practitioners, while also identifying points of tension and difference with both these practices and those within traditional toxicology and model organism biology, which as we will argue appear to be putting precision toxicology on a trajectory to having a separate repertoire from both of these fields.^5^

Reflection on this ongoing transition offers an opportunity to investigate the emergence of what may well turn into a repertoire in real time, while at the same time allowing us to consider various continuities and blurred boundaries between older repertoires and those that emerge by drawing on and adapting them. It also allows exploration of the ways in which emerging repertoires may inspire novel research activities or methodologies, hence providing a framework for analyzing more gradual and distributed shifts in scientific practice. As noted above, the goal in this analysis is not to provide a quantitative metric or similar to determine the precise moment when something becomes a new repertoire, or to provide a definitive checklist of attributes required to make judgments about whether repertoires are the same or different from one another, but instead to consider qualitative factors that contribute adoption, adaptation, and evolution of practices that are associated with a scientific repertoire.

The repertoires approach is markedly different from more traditional philosophical or historical analyses associated with Kuhnian accounts of scientific change (1962) which emphasize normal science and ‘paradigms’ being disrupted by anomalies to the point of ‘revolution’ such that they undergo ‘shifts,’ language that is in common use in scientists’ own accounts of the regulatory changes observed in this space (e.g., Swaters et al. 2022). It also draws on more recent (and alternative historical) accounts in the philosophy and social studies of science of how researchers can and do move between different approaches and models of work depending on a range of circumstances, including making smaller-scale changes and using more than one approach simultaneously (Fleck 1979 [1935]; Giere 2006; Griesemer 2006; Gerson 2013; Nersessian 2022).

The most obvious point of contact between the model organism repertoire and precision toxicology practices are the organisms themselves, both in their material forms as highly domesticated specimens and in their conceptualization as anchors for the repertoire. Precision toxicology is designed to take advantage not only of the highly standardized strains of organisms such as *C. elegans* and *D. melanogaster* that are now widely available from dedicated stock centers (Bangham 2019), but also of the considerable understanding of molecular, physiological, and developmental variation across strains and subspecies that has been accumulated through decades of comparative work on these model species in relation to each other and to humans (Ankeny and Leonelli 2020).

However, there are considerable differences between the model organism repertoire and the emerging practices in precision toxicology, for instance associated with the degree of what we have previously described (writing with Nicole Nelson and Edmund Ramsden) as ‘situatedness’ (Ankeny et al. 2014) of the organisms in these lines of research. In precision toxicology, it is paramount to situate experimental organisms and the research on them within realistic field conditions subject to rapid and unpredictable changes (including the ‘natural experiments’ created by use of model organisms as bioremediators) and understand their reactivity to environmental stressors. Such situatedness, and related ecological and environmental knowledge and sensitivity, is critical to how experimental organisms are used in experiments but also how they are understood and how results are interpreted. In contrast, most more traditional model organism research continues to be lab-based and utilizes a narrow band of strains, with attention remaining centered on the features of the organism under strictly controlled and homogeneous conditions.

The central role allocated to model organisms as well as the differences in how they are conceptualized mark broader overlaps and tensions in the theoretical commitments associated with the model organism repertoire and those emerging in precision toxicology. Similarities include building on evidence of genomic conservation which is used to justify which organisms are selected for use in the first instance, as well as on shared understandings of the organisms’ genomics and resulting phenotypes including physiology and metabolism grounded on decades of relevant research within model organism communities.

However, the use of model organisms in precision toxicology capitalizes specifically on using genomic and other types of data to explore variation, including variable responses to stressors such as potential toxins. Although some more recent model organism research does exploit variability (for instance in ecotype research in Arabidopsis, and recent larger-scale genomic sequencing efforts of diverse strains of *C. elegans* found outside of the lab: see Thompson et al. 2013), precision toxicology does so in tandem with increasing attention to situating the organisms in particular environments that are themselves variable, using a strategy that has been previously identified in some spin-offs of the model organism repertoire (Ankeny and Leonelli 2020). Relatedly, precision toxicology is also deeply interested in how organisms’ metabolic pathways may be expected to react to chemicals within highly unstable environments, thereby directing researchers’ attention to evolving metabolic and other phenotypic processes, as well as to the potential impacts of ecological interactions and the extent to which reactivity to given stressors may be expected to remain stable in complex, real-world conditions.

This difference in conceptual and methodological emphases becomes more evident when we consider the use of model organisms in bioremediation research, where the metabolism of the organisms is instrumentalized and deployed to produce predictable, stable outcomes under highly unstable environmental conditions, but still critically depends on the underlying representational function of the model organisms. The use of organisms as models of toxic effects continues this trend toward the study of environmental sensitivity and variation, while also exploiting the conservation of genomic and evolved pathways in attempts to map and predict organism susceptibility to biochemical stressors on a large scale, once again leveraging the representation function of model organisms. Hence model organisms are simultaneously being used as tools (e.g., indicators for certain chemical concentrations) and due to their representational function (as markers for a specific set of environmental conditions and the ability to project the effects of these conditions onto humans).

Thus a critical distinction between the model organism repertoire and what is emerging in relation to precision toxicology is the epistemic significance assigned to variation, including of the organisms themselves (e.g., minute variations across local strains in particular environmental locations) but also of responses to the environment. This conceptual commitment to detect and track variation is paramount in relation to all three uses of model organisms in practices within precision toxicology. Furthermore, precision toxicology’s emphasis on the power of high-throughput screening for studying such large amounts of variation associated with organisms in particular environmental contexts leverages technologies developed in part during the genomic sequencing projects that were a central contributor to the model organism repertoire but redeploys them to study new objects of interest.

In terms of more institutional and social elements of precision toxicology, large-scale funding has been essential, similar to what occurred with model organisms both individually and in the context of the Human Genome Project in the 1990s. What differs in precision toxicology to date is that funding has not been on the scale that occurred with model organism research once it ramped up, and what has been granted is not as internationalized, with most funding coming solely from the European Union. However these institutional structures are not surprising given that the European Union has been the leading locale for conversion to NAMs (see Ankeny et al. 2024) particularly in regulatory spaces associated with toxicology and the environment (European Union 2010) as well as more fine-grained environmental monitoring including via standard setting (European Commission 2016) and bioremediation strategies. This pattern of funding and support for precision toxicology could rapidly change with increased attention to use of NAMs in the United States, which is a critical regulatory domain, particularly because of the U.S. Environmental Protection Agency’s resolution to phase out mammalian testing by 2035 (EPA 2026) and in light of initiatives by the U.S. Food and Drug Administration (Han 2023; FDA 2025) and National Institutes of Health (Reardon 2025). The shift to NAMs has been further accelerated by the Trump administration using a variety of mechanisms and largely as part of its efficiency measures (Luther 2025).

As is typical in many repertoires, establishment of infrastructures and expertise related to them has been viewed as critical. In part due to relatively limited funding to date, precision toxicology researchers have explicitly called on model organism communities and their infrastructures (such as databases and journals) to support their efforts, for instance by fostering working groups bringing together data scientists, platform managers, and computing specialists from model organism research to discuss the redeployment of their existing tools for use in precision toxicology. An example is the dedicated Toxicology Community launched by ELIXIR in 2018. ELIXIR is an intergovernmental organization that has been supporting resources for life sciences research across Europe for the past decade including model organism work and related infrastructures. The Toxicology Community has the aim of producing a roadmap for how existing tools and standards will need to be modified and adapted to toxicological methods and goals, resulting in a series of recommendations and practical steps for increasing the interoperability and actionability of existing resources (Martens et al. 2023). Such initiatives reveal the dependence of precision toxicology on model organism infrastructures such as databases, classifiers, and computing tools, but also the recognition of the importance of developing and adapting these to serve the needs and goals of precision toxicology itself.

This shift has been furthered by the shared ethos of openness fostered in the model organism repertoire and now taken up by precision toxicology researchers. There is a strong focus on open access and sharing in precision toxicology including of tools and methods, in parallel to this emphasis within the model organism repertoire. This ethos may not seem surprising, given the rapid uptake of such principles growing out of the Human Genome Project and model organism projects within it (Maxson et al. 2018), and the fact that precision toxicology is centered in Europe and the United Kingdom where open science practices are encoded in publication and funding policies. However this focus on openness potentially clashes with the considerable potential for proprietary value to be generated through precision toxicology test kits and methods particularly for environmental screening and via potential products associated with environmental remediation, especially due to the sheer scale of the problems and applications, and hence the potential volume of demand. This feature of the emerging precision toxicology repertoire warrants close monitoring, particularly as results from major projects are disseminated and potentially reused by others in more commercialized settings.

In order to foster openness in the face of possible commodification, precision toxicology advocates have recognized the importance of communication strategies targeting those within the field including toxicologists not utilizing precision approaches as well as scientists from related fields such as developmental biology and even policymakers. These communication strategies are clearly inspired by the extremely effective networking efforts associated with model organism communities and include dedicated websites (PrecisionTox 2024), outreach initiatives, and social media activities. Many of these elements are funded by specific projects such as PrecisionTox and are therefore currently limited in their temporal outlook and scope, but there are focused attempts to build long-term infrastructures and venues for exchange and networking, such as the convening of an annual, stand-alone conference on precision toxicology and the publication of ‘manifestos’ (describing the commitments and goals of precision toxicology) in major scholarly journals (e.g., Coulbourne et al. 2023). These efforts to develop sustainable and transparent modes of communications to ground this distinct research community are a clear mark of the emerging of a precision toxicology repertoire.

A notable difference between the model organism and precision toxicology repertoires consists of the emphasis in the latter on applied and social goals. Such aims were highly limited in model organism case in the short term except in indirect ways, although there were longer-term goals associated with improving human health (later including via precision diagnostics and therapeutics as previously noted). By contrast, precision toxicology is committed to making step changes in the speed, cost, amount, and precision of screening of chemicals and other substances for their impacts on human health and the environment, in part due to the long-standing lack of attention to a huge percentage of substances in toxicology as discussed above.

Here we note the intertwining of different facets of the repertoire, in this case methods and goals, as well as ways in which practices in toxicology are contributing to the precision toxicology repertoire. Toxicologists previously tended to use very simple and highly conservative study designs, typically employing the maximum tolerable dose of the chemical of interest which can be more than 1,000-fold higher than the doses intended for humans (in terms of milligrams per kilogram body weight, for example), resulting in many false positives (Hartung 2009). The turn toward ‘low dose’ toxicology accompanied by risk-benefit assessment (Rietjens and Alink 2006) permitted testing of chemicals in combination and arguably allowed the start of a shift toward new standards and understandings of what counts as evidence in this domain (Hartung 2009). When coupled with precision toxicology’s focus on model organisms and other aspects of its emerging repertoire borrowed from model organisms research, such approaches are permitting rapid short-term outcomes not envisioned previously.

An additional driver that has spurred and shaped the emerging precision toxicology repertoire is the growing awareness of climate change and scientific activities focused on its measurement and remediation of its effects including at larger scale using data-intensive and AI-driven technologies. Precision toxicology emphasizes what is described as ‘embedded translation,’ and from the outset sought to work closely with regulators and other key interested parties in project planning, including selection of chemicals for investigation and case studies for applying precision toxicology-related strategies. This type of embedded translation provides a centerpiece for this emerging repertoire inasmuch as it cements the relationship between investment in new methods and shared social and environmental goals. As with perhaps most repertoires that are being developed at any larger scale, alignment with broader social and political goals is more likely to result in a distinct and sustainable repertoire, and hence the impacts of these factors will be important to trace in coming years.

In summary, similar to the case of the model organism repertoire, use of the same materials via deployment of model organisms alone is not enough to ground a repertoire. Our analysis of the growth of precision toxicology as an emerging potential repertoire underscores the importance of community building; funding structures that are large and coordinated, and ready to recognize this strand of research as promising and worthy of investment; constructing and promoting classifiers and standards for common adoption across relevant research groups; and adequate and relevant infrastructures particularly digitally-based ones such as databases, web platforms, and even social media to foster both collaboration and engagement. Both approaches are grounded in understandings of conservation and projectability, particularly to humans, as key ways in which model organisms function representationally.

At the same time many factors associated with the model organism repertoire and the emerging repertoire of precision toxicology differ significantly: for precision toxicology, its experimental practices are grounded in a specific conceptualization of the organism, particularly with regard to its variability and its roles as deeply situated within realistic ecosystems subject to rapid and unpredictable changes is in stark contrast to the model organism repertoire which relies on strictly controlled and homogeneous conditions largely in laboratory settings. The techniques associated with ‘precision’ are redeployed to explore this variability and the real-life processes at a variety of levels but grounded in the molecular; at the same time, researchers in precision toxicology are interested in contextualizing molecular approaches through evolutionary ecology, thereby using their understanding of gene-environment interactions to foster fundamental thinking around the evolution of trait selection (particularly organisms’ reactivity to toxins). They also diverge in terms of goals and applications, and with respect to the biological interests and expectations underpinning this way of doing research, including the broader social, political, and regulatory drivers that in turn help to shape methods and conceptual understandings underlying these approaches.

## Conclusion

Research programs in the life sciences are utilizing more interdisciplinary methods and borrowing methods, technologies, infrastructures, communication and funding strategies, and other features from different repertoires to address an ever more explicit mandate to understand and mitigate the ongoing climate and biodiversity crises. Arguably these new efforts would not be possible but for the outcomes of the large-scale model organism programs of the last 50 years including sequencing, annotation of function, and technology development. The emergence of precision toxicology including its potential repertoire exemplifies these trends.

This paper has provided an analysis of three uses of model organisms as models in this emerging repertoire that exemplify both continuities and discontinuities with other fields in mid- to late 20th century biology, namely molecular and developmental biology and toxicology itself. In precision toxicology, model organisms are being used for both intervention and representation in ways that are different from the underlying uses originally present in the model organism repertoire, especially in terms of how they facilitate specific forms of environmental action and allow study of variation while still fostering development of deeper evolutionary and developmental understanding. As seen in this extended example, model organisms remain extremely useful and likely central to the future direction of the life sciences. Rather than being primarily used in blue-skies research, they are likely to be increasingly deployed in the context of more applied research methods and approaches which are closely associated with the concept of ‘precision’ including high-throughput approaches, and which accordingly capitalize on diversity, variation, and contextualization. At the same time, these new applied uses may foster novel ways to use model organism research for fundamental biological theorizing, particularly in relation to evolutionary ecology and the emerging understanding of how molecular pathways may have evolved to respond to environmental stressors.

Our analysis of precision toxicology exemplifies how the repertoires framework is a useful heuristic device for tracking the development of a particular scientific approach as a potentially distinctive way of doing research, encompassing a specific epistemic space populated by a well-aligned set of practices, concepts, and instruments (Müller-Wille and Rheinberger 2012) as well as an epistemic collective that acquires a recognizable identity through community building, dedicated funding, and training programs (Knorr Cetina 1999). Considering precision toxicology as a ‘repertoire in the making’ allows identification of the goals and understandings of the concept of ‘precision’ that are becoming to characterize this and related domains. These include attention to variation as a central property of living systems and their environments, thereby studying specific differences in organisms and pathways in response to the environment and stimuli within it; exploitation of high-throughput approaches and technologies that make it possible to document and compare sources and effects of variation; and focus on ecosystem-focused methods, including organisms within situated environments, rather than idealized lab conditions. Given these trends and interests, it is likely that precision toxicology will increasingly turn to what are currently considered to be non-model organisms in the future, ranging from killifish to mosquitos and amphipods (Colbourne et al. 2022), so long as ample baseline genomic information is available. Attention to non-vertebrates is also likely to be spurred on by increased attention to concerns about sentience (New York Declaration on Animal Sentience 2024). For the time being, model organisms continue to play a crucial bridging function precisely due to their value both as representations and as tools for intervention, grounded in decades-long refinement of the model organism repertoire.

Many of the claims in this paper are not limited to the specific case of the relationship between precision toxicology and the model organism repertoire. In a variety of scientific domains, many forms of modelling are critically related to interventions, a role that is likely to become more prominent given the threats presented by global climate change. Consider for instance the use of maps as models, clearly geared to represent a given territory but also to enable orientation and travel across it (whether these are maps of geophysical features, biological pathways, or environmental exposures in a given population), or statistical models that represent specific patterns but also foster certain sets of reactions and interventions, such as the models of transmission frequently used during the COVID-19 pandemic. Analyzing the epistemic details associated with the multiple, often simultaneous, functions of scientific modelling efforts will become ever more relevant given the focus on the complex situatedness of phenomena in the natural world prompted by the climate crisis, including humans’ roles in it.

On a metalevel, this account illustrates the usefulness of the repertoires framework for philosophical explorations of contemporary scientific practices that are still in flux: it permits more nuanced and multifaceted studies of how researchers can and do move between different approaches, models of research, and epistemic understandings, including borrowing from other fields and types of research, depending on a range of factors. The repertoires framework draws on and is in dialogue with approaches from other fields that pay close attention to science and its practices such as history and science and technology studies, but is decidedly grounded in the themes and questions associated with the philosophy of science and particularly science in practice, notably what makes inferences justified, how scientific understandings are developed and deployed, and what the role(s) of models are.

## Data Availability

All data and materials used are in the public domain.

## Footnotes

1 Hence we think it is quite clear that model organisms are not theoretical models, nor are they merely specimens of a larger class of organisms, and thus we do not discuss contrasting views on these points in any detail due to space limitations (cf. Levy and Currie 2015; Parkkinen 2017).

2 Philosophers of science, notably Douglas (2000) and Elliott (2006, 2011), have discussed various instances of so-called ‘regulatory toxicology,’ which is a branch of the broader field of toxicology explicitly devoted to implementing specific ways of detecting, monitoring, and testing for the presence of substances variously defined by regulatory bodies as toxic to humans, and have used these cases as part of the groundwork for their arguments about values in science and particularly decisionmaking related to inductive risk (e.g., Douglas 2009, 2017; Elliott 2017, 2022). Although our arguments have been developed with deep awareness of this literature and its arguments, our explorations go well beyond the narrower field of regulatory toxicology with its distinct features and goals.

3 A historical explanation for the evolution of toxicology as a field would require a paper in and of itself, and thus we do not delve into such an exploration here. Suffice it to say that the ways in which toxicology has been funded, mostly through government-sponsored labs, clinical work on exposed patients, or as part of health and safety research within industry, has encouraged an applied and responsive mode instead of focus on basic research (Davis 2014). The increasing challenges associated with human industrial interventions throughout the 20th century have greatly magnified the challenge of measuring the effects of pollutants in the environment (Landecker forthcoming).

4 For a wealth of references relating to the use of rats as ‘sentinel species’ in toxicology, including in grey literature and toxicological reports, see AI Gemini 2.0 et al. (2025).

5 The repertoires approach also helps to raise the important question of how the precision toxicology repertoire may differ from earlier traditions within toxicological research particularly the well-developed field of regulatory toxicology, and whether it has the features of a repertoire. This question is not one that we can adequately address within the scope of this paper, but warrants future exploration.
